# Sex differences in risk factors for metabolic syndrome in middle-aged and senior hospital employees: a population-based cohort study

**DOI:** 10.1186/s12889-023-15491-4

**Published:** 2023-03-29

**Authors:** Hsu-Chieh Chang, Yi-Syuan Wu, Wen-Chii Tzeng, Hao-Yi Wu, Pai-Ching Lee, Wei-Yun Wang

**Affiliations:** 1 Department of Nursing, Tri-Service General Hospital and School of Nursing, Taipei Medical University, Taipei, Taiwan; 2 Trauma and Critical Care Service, Department of Surgery, Kaohsiung Medical University Hospital, Kaohsiung Medical University, Kaohsiung, Taiwan; 3 School of Nursing, National Defense Medical Center, Taipei, Taiwan; 4 Department of Nursing, Tri-Service General Hospital, Taipei, Taiwan; 5 Department of Nursing, Tri-Service General Hospital and Graduate Institute of Applied Science and Technology, National Taiwan University of Science and Technology, Taipei, Taiwan; 6 Department of Nursing, Tri-Service General Hospital Songshan Branch and School of Nursing, National Defense Medical Center, Taipei, Taiwan; 7 Department of Nursing, Tri-Service General Hospital Songshan Branch, 4F, No. 131, Jiankang Rd., Songshan District, Taipei, 105309 Taiwan, ROC

**Keywords:** Hospital employees, Sex difference, Metabolic syndrome, Middle-aged, Senior, Lifestyle habit

## Abstract

**Background:**

Several cross-sectional studies have reported risk factors for metabolic syndrome (MetS). However, these studies did not focus on sex differences in middle-aged and senior populations or employ a longitudinal design. These study design differences are important, as there are sex differences in lifestyle habits associated with MetS, and middle-aged and senior individuals have increased MetS susceptibility. Therefore, the purpose of this study was to examine whether sex differences influenced MetS risk over a ten-year follow-up period among middle-aged and senior hospital employees.

**Methods:**

This population-based and prospective cohort study enrolled 565 participants who did not have MetS in 2012 for a ten-year repeated-measurement analysis. Data were retrieved from the hospital’s Health Management Information System. Analyses included Student’s *t* tests, χ^2^ tests and Cox regression. *P* < 0.05 indicated statistical significance.

**Results:**

Male middle-aged and senior hospital employees had an elevated MetS risk (hazard ratio (HR) = 1.936, p < 0.001). Men with more than four family history risk factors had an increased risk of MetS (HR = 1.969, p = 0.010). Women who worked shift duty (HR = 1.326, p = 0.020), had more than two chronic diseases (HR = 1.513, p = 0.012), had three family history risk factors (HR = 1.623, p = 0.010), or chewed betel nuts (HR = 9.710, p = 0.002) had an increased risk of MetS.

**Conclusions:**

The longitudinal design of our study improves the understanding of sex differences in MetS risk factors in middle-aged and senior adults. A significantly elevated risk of MetS over the ten-year follow-up period was associated with male sex, shift work, the number of chronic diseases, the number of family history risk factors, and betel nut chewing. Women who chewed betel nuts had an especially increased risk of MetS. Our study indicates that population-specific studies are important for the identification of subgroups susceptible to MetS and for the implementation of hospital-based strategies.

## Background

Metabolic syndrome (MetS) is a complex pathophysiologic condition characterized by several markers of dysregulation, including obesity, hypertension, hyperlipidemia, and elevated blood glucose levels, and is a strong predictor of morbidity and cardiovascular disease (CVD)-related mortality [1, 2]. Previous studies have indicated that early stage prevention and intervention are very effective at preventing and reducing the severity of CVD [3, 4]. The middle-age and senior periods are important stages in the prevention and management of MetS owing to the increased susceptibility of individuals to MetS during these periods [5–7]. The risk of cardiovascular disease is notably increased in middle-aged and senior adults with MetS [8], and as many as 50–60% of older adults have been reported to meet the criteria for MetS [9, 10]. In addition, middle-aged and senior hospital employees played an important role in hospital management and inheritance of experience. If they had health problems, it would prevent employees from completely engaging in work activities and diminish their workload [11, 12]. Therefore, health professionals should attempt to reduce the development of MetS in middle-aged and senior individuals.

Hospital employees have better health knowledge than the general public, but because they face the high-pressure environment of life and death, hospital employees are prone to illness [13]. In addition, the nature of the hospital setting requires work in alternating shifts, which increases the vulnerability of hospital employees. The disruption of circadian rhythms among shift employees is proposed to lead to loss of internal synchrony, and disrupted sleep-wake cycles and metabolic pathways have been linked to an increased risk of MetS [14, 15]. In hospital employees, MetS impacts health, which is related to work productivity. Productivity is reduced when employees are ill or have health problems that reduce their commitment to work activities [11, 12], which can also have a major impact on the fiscal performance of the institution [16]. Therefore, the prevention of MetS in hospital employees is an important issue.

Previous studies have identified numerous lifestyle habits associated with MetS, including diet [17, 18], cigarette smoking [19], alcohol consumption [20] and betel nut chewing [21]. In addition, the presence of a family history of risk factors and chronic diseases [22] were correlated with MetS. Demographic characteristics, such as being male [23–25] and middle-aged and older adults [21] are also risk factors for MetS. Moreover, lifestyle habits associated with MetS also exhibit sex differences [19, 26, 27]. Therefore, research is needed to evaluate sex differences in lifestyle habits related to MetS.

However, most of the studies on MetS have employed a cross-sectional design and few have examined MetS and its risk factors in middle-aged and senior hospital employees with a longitudinal cohort. Therefore, the purpose of this study was (1) to examine whether there were sex differences in lifestyle habits that contribute to the risk of MetS and (2) to identify possible sex differences in risk factors for MetS during ten years of follow-up among middle-aged and senior hospital employees.

## Methods

### Design

A population-based and prospective longitudinal cohort study was conducted.

### Study population

**Fig. 1:**
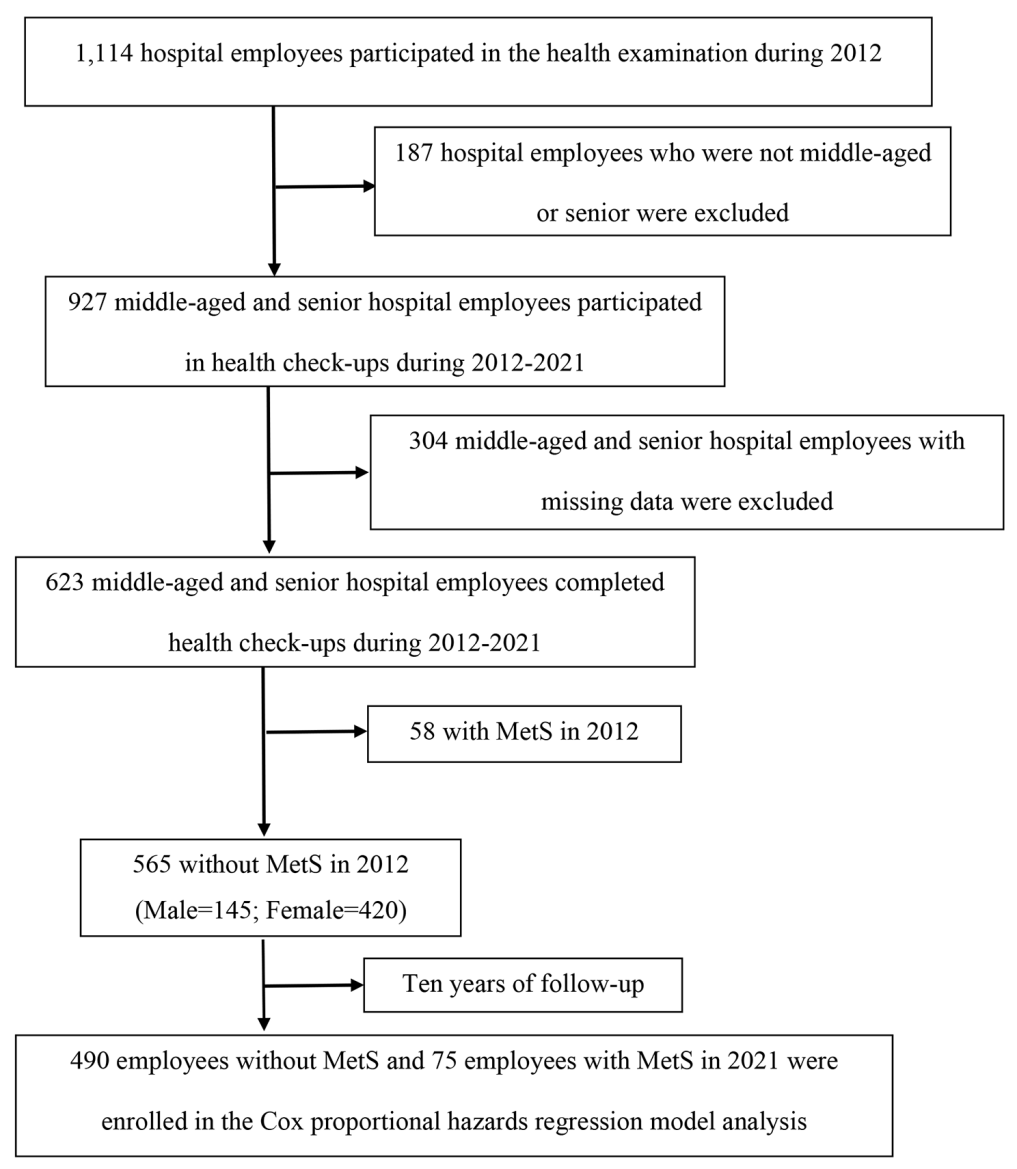
Flow chart of study participant enrollment

We used data from the hospital’s Health Management Information System (HMIS) during 2012–2021. The HMIS was established in 2012 and includes annual worksite health check-ups of hospital employees. A total of 1,114 hospital employees participated in the health examination during 2012. In total, 187 hospital employees who were not middle-aged or senior were excluded. Moreover, 927 middle-aged and senior hospital employees participated in health check-ups during 2012–2021. Next, 304 employees who had missing health check-up data were excluded. This left a total of 623 middle-aged and senior hospital employees who had completed health check-ups within the period of 2012–2021. The 565 middle-aged and senior hospital employees who were MetS-free in 2012 and underwent ten repeated health assessments were further analyzed. After ten years of follow-up, 75 of these participants were diagnosed with MetS in 2021. The flow chart of study participant enrollment is shown in Fig. 1.

### Data collection

Data from HMIS participants were collected through a questionnaire and annual worksite health examinations, including anthropometric and biochemical data. Detailed information on the data collection is described elsewhere [25]. In our analyses of demographic and lifestyle habits, we used date of birth, sex, shift work, drinking milk, eating sufficient vegetables and fruits each day, smoking status, alcohol consumption, and betel nut chewing. Regarding medical histories, number of chronic diseases and number of family history risk factors were assessed. Family history risk factors included a family history of diabetes mellitus, hypertension, heart disease, or stroke in at least one parent. Demographic characteristics, lifestyle habits and medical histories were provided in a self-report questionnaire. All participants provided informed consent prior to participation in this study, and their data from questionnaires and annual worksite health examinations was input into the HMIS. To maintain personal privacy, all identification in the database was encrypted. This study was conducted according to principles of the 1975 Declaration of Helsinki and was approved by the institutional review board of the Tri-Service General Hospital in Taiwan (approval number: A202005134).

### Data characteristics and diagnostic criteria

Middle-aged adults were defined as adults who were 31–50 years old, and senior adults were defined as adults who were 51–64 years old [28]. We used the National Cholesterol Education Program Adult Treatment Panel III [29] diagnostic criteria for MetS and modified the International Diabetes Federation’s definition accounting for waist circumstance norms in the Asian population.

The five indicators of metabolic dysregulation are elevated fasting plasma glucose (FPG level ≥ 100 mg/dL), elevated triglycerides (TG level ≥ 150 mg/dL), lowered high-density lipoprotein cholesterol (HDL-C) level (< 50 mg/dL in women or < 40 mg/dL in men), elevated blood pressure (systolic blood pressure (SBP) ≥ 130 mmHg or diastolic blood pressure (DBP) ≥ 85 mmHg), and an increased waistline (waist circumstance (WC) ≥ 80 cm in women or ≥ 90 cm in men). If the participants met ≥ 3 of any of the 5 indicators, they were defined as having MetS [30].

### Data analysis

Participants were categorized into 2 groups according to the presence or absence of MetS. The MetS group consisted of participants who met 3 or more of the 5 criteria for MetS. The non-MetS group comprised participants who met 2 or fewer criteria for MetS. The distributions of demographic characteristics, lifestyle habits and medical histories of the two groups were compared according to the presence or absence of MetS. Student’s *t* tests were used to compare continuous variables between groups, whereas χ^2^ tests were used to compare categorical variables. The 565 middle-aged and senior hospital employees who were MetS-free in 2012 and underwent ten repeated health assessments were further analyzed. A cox proportional hazards regression model was used to evaluate the associations between demographic characteristics, lifestyle habits and medical histories and the development of MetS. All data management and statistical analyses were performed using SPSS (version 27.0, SPSS Inc., Chicago, IL, USA). Statistical significance was set at p < 0.05, and a 95% confidence interval was employed.

## Results

### Characteristics of participants with and without MetS

In this study, we included data from a total of 565 middle-aged and senior hospital employees collected from 2012 to 2021; 145 participants were male, and 420 were female. The mean age was 46.80 years (standard deviation = 6.97). A total of 490 middle-aged and senior hospital employees were MetS-free and 75 had MetS in 2021. Sex (χ^2^ = 4.843, p = 0.028), smoking status (χ^2^ = 6.133, p = 0.013), betel nut chewing (χ^2^ = 5.388, p = 0.020), the number of chronic diseases (χ^2^ = 15.093, p = 0.001), and the number of family history risk factors (χ^2^ = 20.881, p < 0.001) significantly differed between groups with and without MetS (Table 1).

**Table 1:** Demographic characteristics, lifestyle habits and medical histories related to metabolic syndrome (N = 565)

Variable	Total (N = 565)	Metabolic syndrome		χ^2^ / t	p
		No (N = 490)	Yes (N = 75)		
Age (years)	46.80 ± 6.97^a^	46.65 ± 6.92^a^	47.84 ± 7.28^a^	-1.371	0.171
Sex				4.843	0.028
Male	145 (25.7)	118 (81.4)	27 (18.6)		
Female	420 (74.3)	372 (88.6)	48 (11.4)		
Shift work				1.863	0.172
Yes	305 (54.0)	270 (88.5)	35 (11.5)		
No	260 (46.0)	220 (84.6)	40 (15.4)		
Drinks milk				0.019	0.889
Yes	21 (3.7)	18 (85.7)	3 (14.3)		
No	544 (96.3)	472 (86.8)	72 (13.2)		
Eats at least three servings of vegetables and two servings of fruit per day				1.169	0.280
Yes	224 (39.6)	190 (84.8)	34 (15.2)		
No	341 (60.4)	300 (88.0)	41 (12.0)		
Smoking				6.133	0.013
Yes	23 (4.1)	16 (69.6)	7 (30.4)		
No	542 (95.9)	474 (87.5)	68 (12.5)		
Drinks alcohol				1.417	0.234
Yes	156 (27.6)	131 (84.0)	25 (16.0)		
No	409 (72.4)	359 (87.8)	50 (12.2)		
Chews betel nuts				5.388	0.020
Yes	7 (1.2)	4 (57.1)	3 (42.9)		
No	558 (98.8)	486 (87.1)	72 (12.9)		
Number of chronic diseases				15.093	0.001
≥ 2	59 (10.5)	43 (72.9)	16 (27.1)		
1	151 (26.7)	127 (84.1)	24 (15.9)		
0	355 (62.8)	320 (90.1)	35 (9.9)		
Number of family history risk factors				20.881	< 0.001
≥ 4	38 (6.7)	24 (63.2)	14 (36.8)		
3	78 (13.8)	66 (84.6)	12 (15.4)		
2	132 (23.4)	117 (88.6)	15 (11.4)		
1	164 (29.0)	147 (89.6)	17 (10.4)		
0	153 (27.1)	136 (88.9)	17 (11.1)		

Values indicate counts (percentages) unless stated otherwise

^a^ Mean ± standard deviation

## Risk factors for MetS in middle-aged and senior hospital employees

After ten years of follow-up, 75 of the 565 individuals were diagnosed with MetS in 2021; the prevalence rate was 13.27%. The risk factors for MetS in middle-aged and senior hospital employees are shown in Table 2. After controlling for smoking status, alcohol consumption and chewing betel nuts, the variables of sex, shift work, the number of chronic diseases, and the number of family history risk factors were significantly associated with the risk of MetS. Middle-aged and senior hospital employees who were male (hazard ratio (HR) = 1.936, p < 0.001), had shift work duty (HR = 1.204, p = 0.047), had more than two chronic diseases (HR = 1.399, p = 0.012), had three family history risk factors (HR = 1.579, p = 0.001), or had more than four family history risk factors (HR = 1.571, p = 0.008) had a significantly elevated risk of MetS.

**Table 2:** Demographic characteristics, lifestyle habits and medical histories associated with the risk of metabolic syndrome (N = 75)

Variable	Beta	p value	Hazard ratio (95% CI)
Sex			
Male	0.661	< 0.001	1.936 (1.586 ~ 2.363)
Female ^a^			
Shift work			
Yes	0.185	0.047	1.204 (1.002 ~ 1.446)
No ^a^			
Number of chronic diseases			
≥2	0.335	0.012	1.399 (1.075 ~ 1.819)
1	0.182	0.077	1.199 (0.980 ~ 1.467)
0 ^a^			
Number of family history risk factors			
≥4	0.452	0.008	1.571(1.123 ~ 2.196)
3	0.457	0.001	1.579 (1.192 ~ 2.093)
2	0.101	0.451	1.107 (0.850 ~ 1.440)
1	-0.098	0.453	0.906 (0.701 ~ 1.172)
0 ^a^			
Smoking			
Yes	-0.041	0.859	0.960 (0.610 ~ 1.509)
No ^a^			
Drinks alcohol			
Yes	0.028	0.793	1.029 (0.833 ~ 1.270)
No ^a^			
Chews betel nuts			
Yes	0.465	0.233	1.592(0.742 ~ 3.417)
No ^a^			

Abbreviations: CI, confidence interval. ^a^ Reference group

## Sex differences in characteristics associated with the risk of MetS

The variable of sex was significantly associated with the risk of MetS. Middle-aged and senior hospital employees who were male had a significantly elevated risk of MetS. In men, having more than four family history risk factors (HR = 1.969, p = 0.010) significantly increased the risk of MetS. In women, shift work duty (HR = 1.326, p = 0.020), having more than two chronic diseases (HR = 1.513, p = 0.012), having three family history risk factors (HR = 1.623, p = 0.010), and chewing betel nuts (HR = 9.710, p = 0.002) significantly increased the risk of MetS (Table 3).

**Table 3:** Demographic characteristics, lifestyle habits and medical histories associated with the risk of metabolic syndrome by sex (N = 75)

	Men (N = 27)			Women (N = 48)			
Variable	Beta	p value	Hazard ratio (95% CI)	Beta	p value	Hazard ratio (95% CI)	
Shift work							
Yes	0.038	0.804	1.039 (0.770 ~ 1.400)	0.282	0.020	1.326 (1.046 ~ 1.681)	
No ^a^							
Number of chronic diseases							
≥2	0.155	0.507	1.167 (0.739 ~ 1.844)	0.414	0.012	1.513 (1.097 ~ 2.086)	
1	0.283	0.103	1.327 (0.945 ~ 1.865)	0.110	0.392	1.116 (0.868 ~ 1.435)	
0 ^a^							
Number of family history risk factors							
≥4	0.678	0.010	1.969 (1.174 ~ 3.303)	0.355	0.127	1.426 (0.904 ~ 2.249)	
3	0.429	0.085	1.536 (0.942 ~ 2.505)	0.484	0.010	1.623 (1.123 ~ 2.345)	
2	0.009	0.970	1.009 (0.644 ~ 1.580)	0.162	0.365	1.175 (0.829 ~ 1.666)	
1	-0.219	0.261	0.804 (0.549 ~ 1.177)	-0.020	0.915	0.981 (0.682 ~ 1.409)	
0 ^a^							
Smoking							
Yes	-0.105	0.676	0.900 (0.551 ~ 1.473)	0.055	0.940	1.057 (0.252 ~ 4.428)	
No ^a^							
Drinks alcohol							
Yes	0.232	0.205	1.262 (0.880 ~ 1.808)	-0.056	0.676	0.946 (0.727 ~ 1.229)	
No ^a^							
Chews betel nuts							
Yes	0.166	0.710	1.180 (0.492 ~ 2.829)	2.273	0.002	9.710 (2.307 ~ 40.874)	
No ^a^							

Abbreviations: CI, confidence interval. ^a^ Reference group

## Discussion

Early exposures increase an individual’s risk of MetS throughout the lifespan. The middle-aged and senior periods are important due to the increased susceptibility of MetS. In addition, healthcare providers in Taiwan graduated from school at 20–25 years old, then started working as hospital employees. Therefore, the employees who were under 30 years old were relative novice and learning was their main task. However, the employees who were over 30 years old were relative stable and played an important role in the hospital management and inheritance of experience. If they had health problems, it would prevent employees from completely engaging in work activities and diminish their workload. This has a negative impact on hospital management. Therefore, we investigated the risk factors for MetS in middle-aged and senior adults to prevent MetS as soon as possible. To our knowledge, this study is one of the few to investigate sex differences in the risk factors for MetS in middle-aged and senior adults with a longitudinal cohort design.

Our study yielded four main findings. First, sex differences existed. Our study revealed that, compared with women, men were at a 1.936-fold greater risk of developing MetS, which was not consistent with a previous study [31]. This finding may be because the majority of our subjects were middle-aged women. Middle-aged women may benefit from exposure to female hormones, especially estrogen, which inhibit fibrogenesis, promote antioxidant effects, increase innate immunity, inhibit cellular senescence, protect mitochondrial structure and function, protect against visceral fat accumulation by decreasing serum low-density lipoprotein-cholesterol (LDL-C), increase high-density lipoproteins, and inhibit coronary thrombosis and atherosclerosis by regulating vascular smooth muscle and endothelial cells [16, 32–34]. Thus, the majority of the subjects in our study were middle-aged women, which resulted in their lower risk of developing MetS than the male subjects. Sex influenced health and wellbeing in a variety of ways [35]. For instance, men are socially conditioned to neglect pain and disease, resulting in a general under-utilization of health services and a lower likelihood to engage in routine check-ups compared with women [36]. Women are more health conscious than men and immediately seek medical counsel or an examination in the event of physical discomfort; therefore, the risks of chronic diseases are usually lower in women than in men [13, 16, 34]. When women have chronic diseases, it represented their overall health declines [37]. Similarly, our study showed that women with more than two chronic diseases had a 1.513-fold greater risk for developing MetS.

Our study not only showed that men were at greater risk for developing MetS, but we further noted that women who had a habit of chewing betel nuts had an HR of 9.710 (95% confidence interval (CI): 2.307–40.874) for MetS. The links between betel nut chewing and MetS in women might be related to health consciousness and increased oxidative stress, inflammation, adipogenesis and appetite [38–42]. Because women usually are more health conscious and do not use hazardous substances, women with a habit of chewing betel nuts may be less health conscious and more likely to engage in activities that are hazardous to their health [43]. At the same time, betel nuts may induce reactive oxygen species (ROS) production, cell cycle aberrations and irregular cell differentiation, and platelet aggregation and increase the lipid profile burden by increasing oxidative stress and inflammation. In addition, hydroxychavicol, areca alkaloids and arecoline compounds found in betel nuts inhibit the differentiation of adipose tissue and induce adenylyl-cyclase-dependent lipolysis, both of which contribute to hyperlipidemia. Moreover, areca alkaloids also stimulate appetite via the inhibition of gamma-amino butyric acid (GABA) receptors; an established betel nut chewing habit thus increases appetite, which contributes to the risk of obesity [21, 38, 44]. In this manner, betel nut chewing in women increased the risk of MetS.

There is a sex dimension to this, with sex hormones, health awareness and preventable risk factors accounting for the prevalence of MetS. Sex affects physiological risks, access to health care, health-seeking behavior, health care utilization, and risk-taking behaviors, thus impacting the variation of MetS prevalence in middle-aged and senior hospital employees. In the future, not only sex but also gender should be considered throughout the research process, from the design of research questions to the interpretation of study results, with segregation of results by sex or gender.

Second, lower MetS prevalence existed in middle-aged and senior adults among hospital employees. According to our study, the prevalence of MetS in middle-aged and senior hospital employees was similar to that of other hospitals [33, 45] and was lower than that in the general population [13]. This may be attributed to the Health Promoting Hospital (HPH) concept, which was promulgated by the World Health Organization (WHO) in 1998. Adhering to the HPH trend, the Taipei Department of Health established a “Healthy Hospital Accreditation” (HHA) in 2002 to encourage hospital managers to organize medical resources that promoted the health of employees. The HHA emphasized occupational health, such as the promotion of work efficiency, healthy living habits, cessation of smoking, control of body weight, and physical fitness [13, 16]. Therefore, the health of hospital employees is a matter of great concern for hospital management. Hospitals need ensure the health of employees to expand the influence of health care services. Thus, hospital employees can easily access medical services or healthier lifestyle habits. In addition, most of the hospital employees with higher education levels might have better health knowledge, which could be helpful in maintaining healthy behaviors, preventing MetS, and seeking professional medical treatment, all of which positively influence metabolic health among hospital employees [46].

Another possible explanation for the low prevalence of MetS in hospitals might be due to the “healthy worker effect” found by other studies [16, 47]. The employees underwent a preemployment screening for health and were routinely selected from young and strong individuals. However, our study focused on middle-aged and senior hospital employees who were 31–65 years old and excluded young hospital employees. In addition, during the middle- age and senior periods, people experience an increased risk of CVD and susceptibility to MetS [5–8]. This showed that the promotion of the HHA increased the metabolic health of our hospital employees in the past years.

The middle-age and senior periods are important stage in the prevention and management of MetS owing to the increased susceptibility of individuals to MetS during these periods [5–7]. In addition, the risk of CVD is notably increased in middle-aged and senior adults with MetS [8]. As many as 50–60% of senior workers have been reported to meet the criteria for MetS [9, 10]. This may be due to unhealthy behaviors like betel nut chewing, which are assumed to play a causal role in MetS incidence and might have accumulated over the life course, leading to a stronger detrimental health effect among senior workers [48]. Therefore, health professionals should attempt to reduce the development of MetS and CVD in middle-aged and senior hospital employees. In the future, life-course studies with clinical outcomes such as MetS and CVD events are needed to determine the possible cumulative influence of early life health behaviors and work history on later life health (dis-)advantages.

Third, genetic factors may play a role in developing MetS. Our study showed that having three or more family history risk factors was associated with a significantly elevated risk of MetS among middle-aged and senior hospital employees. Paek et al. [49] found that a family history of hypertension/stroke is associated with a statistically significant increased risk of MetS in both men and women. Yeboah et al. [50] showed that a positive history of parental CVD was associated with an increase in the risk of MetS [OR (95% CI): 1.23 (1.12–3.04), p = 0.037]. Chiu et al. [51] also showed that a family history of DM was significantly associated with MetS. In addition, individuals with both a sibling and parental family history of DM had the highest prevalence of MetS. MetS is a complex pathophysiologic condition that originates primarily from an imbalance in calorie intake and energy expenditure but is also affected by genetic factors [1, 52]. From these research findings, we can see the scope of genetic influence on MetS in the familial history.

Fourth, longitudinal cohort design made up the knowledge gap of MetS among the aging hospital workforce. The results of the Cox proportional hazard regression model showed that sex, shift work, the number of chronic diseases, and the number of family history risk factors were associated with a significantly elevated risk of MetS in our study. Further, more research should be done with longer and repeated follow-up data from several assessment waves and consider the influence of changes in health behaviors over time.

While this study offers significant contributions to the current literature on the metabolic health of middle-aged and senior hospital employees, it also presents limitations. First, the HMIS contained limited information on potentially confounding factors (e.g., job category, marriage age, medication history, reproductive history, physical activity, mental health status) and lacked dietary subdivisions. Second, it was a second-hand dataset containing self-reported information, which may have resulted in bias, such as recall bias. Third, the results cannot be generalized because this study focused on the employees of only one hospital. Although we utilized a ten-year longitudinal design to follow-up with the cohort, our participants included considerably fewer men (26%) than women (74%). Compared to the other studies, there was also a similar distribution of sex ratios in the hospital staff. The male vs. female ratio of hospital employees is 24.7% vs. 75.3% in Italy [53], 11.1% vs. 88.9% in China [54], 22.2% vs. 77.8% in USA [55], 13% vs. 87% in Finland [56], 24% vs. 76% in France [57], and 32.5% vs. 67.5% in Saudi Arabia [58]. Although there were fewer male than female participants in our study, male participants had a significantly elevated risk of MetS. Our results also could be better presented as predictors of developing MetS in female hospital employees. Additional studies should be conducted in a clinical setting to overcome these limitations and to investigate the lifestyle patterns, job category, marriage age, medication history, reproductive history, physical activity and mental health status of hospital employees.

## Conclusions

Our study provides a better understanding of MetS and sex differences in its risk factors in a longitudinal cohort of middle-aged and senior hospital employees. We found that the following factors were associated with a significantly elevated risk of MetS over the ten years of follow-up: male sex, having shift work duty, the number of chronic diseases, and family history of risk factors. Female subjects who had a habit of chewing betel nuts had an increased risk of MetS. The sex-specific risk factors for MetS in middle-aged and senior hospital employees were identified through health examinations. Our study indicates that population-specific studies are important for identifying subgroups susceptible to MetS and for implementing hospital-based strategies.

## Data Availability

The datasets used and/or analyzed during the current study are available from the corresponding author on reasonable request.

## Abbreviations

MetS: Metabolic syndrome
CVD: Cardiovascular disease
WC: Waist circumstance
SBP: Systolic blood pressure
DBP: Diastolic blood pressure
FPG: Fasting plasma glucose
TG: Triglyceride
HDL-C: High density lipoprotein cholesterol
HMIS: Health Management Information System
HR: Hazard ratio
CI: Confidence interval
LDL-C: Lowdensity lipoprotein-cholesterol
ROS: Reactive oxygen species
GABA: Gamma-amino butyric acid
HPH: Health-Promoting Hospital
WHO: World Health Organization
HHA: Healthy Hospital Accreditation.
